# Rapid Bacterial Identification and Quantitative Antimicrobial Susceptibility Assessment from Positive Blood Cultures to Optimize Bloodstream Infection Management

**DOI:** 10.3390/microorganisms14030633

**Published:** 2026-03-11

**Authors:** Lucia Sliviaková Matúšková, Michala Vladárová, Elena Nováková

**Affiliations:** 1 Department of Clinical Microbiology, Clinical Biochemistry Ltd., 01007 Žilina, Slovakia; 2 Institute of Microbiology and Immunology, Jessenius Faculty of Medicine in Martin, 03601 Martin, Slovakia

**Keywords:** bloodstream infection, blood cultures, MALDI-TOF MS, antibiotic susceptibility testing, rapid microbiological diagnostics, treatment of BSI

## Abstract

Bloodstream infection (BSI) is a serious clinical condition associated with high morbidity and mortality, requiring rapid identification of causative agents and timely antimicrobial susceptibility testing (AST). This study evaluated accelerated bacterial identification from positive blood culture samples using matrix-assisted laser desorption/ionization time-of-flight mass spectrometry (MALDI-TOF MS) combined with two rapid processing approaches: a serum separation tube-based centrifugation method (SST method) and shortened cultivation on solid media. Rapid identification was followed by accelerated AST, performed either from a bacterial cell pellet (SST method) and from early-grown bacterial biomass (shortened cultivation protocol). The results were compared with those obtained using routine laboratory procedures. A total of 270 positive blood culture samples were analyzed, with 135 samples processed by each protocol. Both approaches achieved an identification success rate of 93.33%. Rapid AST using the SST method showed error rates of 0.51% minor errors, 0.57% major errors, and 0.23% very major errors, with an overall agreement of 98.69%. The shortened cultivation protocol demonstrated lower error rates (0.46% minor errors and 0.23% major errors) and an overall agreement of 99.31%. These findings confirm that MALDI-TOF MS enables reliable early identification of BSI pathogens and rapid AST, supporting timely optimization of antimicrobial therapy and early detection of multidrug-resistant strains.

## 1. Introduction

Blood culture collection represents the gold standard for the diagnosis of bloodstream infections (BSIs) and septic conditions. BSIs are associated with high morbidity and mortality, and timely microbiological diagnosis is essential for guiding appropriate therapy [1,2]. Between blood culture collection and pathogen identification with antimicrobial susceptibility testing (AST), a diagnostic “window of uncertainty” exists, during which patients may receive inappropriate empirical therapy, negatively impacting in early stages of infection [3]. Rapid identification of bacterial pathogens from positive blood cultures, together with accelerated determination of pathogen-specific antimicrobial susceptibility, is critical for effective patient management within the framework of antimicrobial stewardship.

Matrix-assisted laser desorption/ionization time-of-flight mass spectrometry (MALDI-TOF MS) enables rapid identification of bacterial pathogens from positive blood cultures. While standard workflows require subculture for 16–24 h (up to 72 h for anaerobic organisms) before identification, direct MALDI-TOF MS or shortened subculture approaches allow same-day pathogen identification, significantly accelerating targeted therapy [4,5].

Rapid AST is equally critical for antimicrobial stewardship, allowing prompt initiation or de-escalation of therapy and reducing the risk of resistance. Accelerated AST can shorten the time to results by up to 24 h, supporting more effective and timely management of patients with BSI [6].

**Figure 1:**
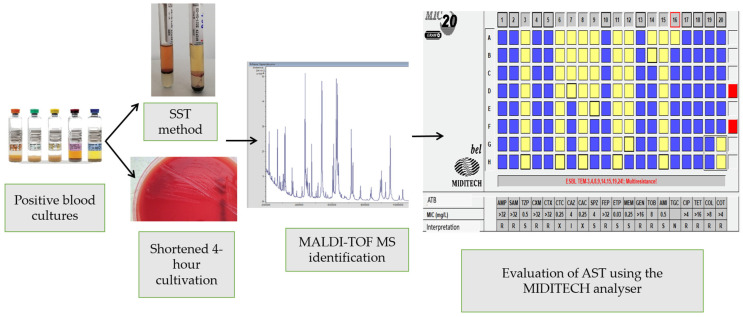
Overview of the study workflow.The figure presents a schematic illustration of the sequential steps of the study procedure, from sample processing with two protocols (SST method and shortened cultivation) following rapid identification by MALDI-TOF MS to final AST through Bel-MIDITECH diagnostic AST panel and interpretation of the results using the MidiTech Analyser.

The aim of this study was to evaluate the effectiveness of rapid pathogen identification from positive blood cultures using MALDI-TOF MS, performed either directly by the SST method or after shortened incubation on solid media. The study further aimed to assess the contribution of rapid AST and to determine whether the susceptibility results obtained with this approach are consistent with those obtained using standard AST procedures (Figure 1). Finally, the study aimed to evaluate the impact of these rapid approaches on reducing the time to microbiological diagnostics.

## 2. Materials and Methods

Positive blood cultures processed at the Department of Clinical Microbiology, local microbiological laboratory in Slovak republic, between October 2024 and June 2025 were included in the study. Samples were obtained from patients hospitalized in different departments of the University Hospital with polyclinic in Žilina. Blood cultures were incubated in the automated BacT/ALERT 3D system (bioMérieux, Marcy-l’Étoile, France) until positivity was detected.

Positive blood cultures were processed according to the standard laboratory workflow, including Gram staining and subculture on solid media for 16–24 h at 37 °C in a 5% CO_2_ atmosphere. Species identification was performed by MALDI-TOF MS from freshly grown colonies using a MALDI Microflex LT instrument (Bruker, Leipzig, Germany). Mass spectra were acquired in positive linear mode (*m*/*z* 2000–20,000) using a nitrogen laser (λ = 337 nm) and analyzed with MALDI BioTyper Compass software v4.1.100 (Bruker, Leipzig, Germany). Routine AST was performed after 16–24 h of cultivation using a modified broth microdilution method (Bel-MIDITECH s.r.o., Bratislava, Slovakia) or gradient diffusion test (bioMérieux, Marcy-l’Étoile, France), requiring an additional 16–24 h for result interpretation. In the modified broth microdilution method, we followed the procedure provided by the manufacturer; the detailed protocol is described further in the text.

In our study, the modified broth microdilution method was performed using a diagnostic AST panel manufactured by Bel-MIDITECH s.r.o. (Bratislava, Slovakia), certified for in vitro AST in accordance with applicable standards. The diagnostic AST panel enables quantitative susceptibility assessment (minimum inhibitory concentrations, MICs determination) for *Enterobacteriaceae*, Gram-negative non-fermenting bacteria, staphylococci, and *Enterococcus faecalis* against a broad range of clinically relevant antimicrobial agents. The principle of determination corresponds to the reference broth microdilution method for AST. The diagnostic AST panel employs a colorimetric metabolic indicator system to assess bacterial growth following antibiotic exposure. The reaction wells of the diagnostic AST panel contain predefined concentrations of antibiotics, as indicated in the Appendix A (Table A1, Table A2, Table A3 and Table A4). Briefly, a bacterial inoculum equivalent to 0.5 McFarland is prepared in sterile physiological saline (Sodium chloride, Slavus, Slovakia and distilled water). The suspension is subsequently transferred into Mueller–Hinton broth (CM0405B, Thermo Fisher Scientific, Massachusetts, USA) to achieve a final inoculum density of 0.5–1 × 10^6^ CFU/mL, which was then used to inoculate the reaction wells. The inoculated reaction wells are incubated at 37 °C for 16–20 h in a sealed plastic bag. Following incubation, the diagnostic AST panel are stained with a growth indicator (0.08% 3-(4,5-dimethylthiazol-2-yl)-2,5-diphenyl tetrazolium bromide; Bel-MIDITECH s.r.o., Bratislava, Slovakia) and incubated for an additional 30 min at 37 °C (60 min for *Enterococcus faecalis*). The results are read immediately after staining or, alternatively, the diagnostic AST panel may be frozen for subsequent analysis. Interpretation is performed according to EUCAST or CLSI breakpoint criteria, as appropriate. Automated photometric reading is carried out using the MidiTech Analyser expert system (Bel-MIDITECH s.r.o., Bratislava, Slovakia), which incorporates a microbiological expert system and a pharmacological consultation module and can be integrated with the laboratory information system. The accuracy of MIC determination was verified by internal quality control using reference strains with established MIC ranges for the broth microdilution method: *Escherichia coli* ATCC 25922, *Pseudomonas aeruginosa* ATCC 27853, *Staphylococcus aureus* ATCC 29213, and *Enterococcus faecalis* ATCC 29219.

To accelerate identification, direct MALDI-TOF MS identification from positive blood cultures was performed using the serum separator tube centrifugation method (SST method; using SST tube-Vacutest clot). After obtaining the bacterial pellet, the sample was further processed for MALDI-TOF MS identification using an extraction step with ethanol and formic acid. In parallel, an accelerated subculture approach was applied, in which positive blood culture samples were inoculated onto blood agar and incubated for 4 h at 37 °C in a 5% CO_2_ atmosphere. Identification was performed from bacterial biomass obtained after 4 h of incubation. Samples were analyzed in duplicate using standard HCCA matrix preparation.

An in-house MALDI-TOF MS score cut-off of ≥1.5 was applied, with consistency of results taken into account. Identification accuracy was verified by conventional MALDI-TOF MS identification from overnight subcultures. Isolates yielding unreliable mass spectra or no identification were classified as unidentifiable (no organism identified peak, NOIP).

To accelerate AST, quantitative susceptibility testing was performed using the modified broth microdilution method for both accelerated approaches. A standardized inoculum equivalent to 0.5 McFarland was prepared either from the bacterial pellet obtained during SST processing or from bacterial biomass grown after 4 h of subculture. MICs were interpreted according to EUCAST criteria using the MIDITECH Analyser.

## 3. Results

### 3.1. Samples Tested

For the purpose of this study, 135 positive blood cultures were processed using the SST method and an additional 135 samples using the shortened cultivation method on blood agar. Blood culture samples with polymicrobial growth were excluded from the analysis.

Identification of clinical isolates was initially assessed according to the manufacturer-recommended MALDI-TOF MS cut-off values. However, identifications with MALDI scores < 1.7 were also considered valid if consistent results were obtained. For MALDI scores ≤1.699, identification was accepted based on consistent results in the MALDI BioTyper Compass 4.1.100 software and a study-specific cut-off of ≥1.5. The accuracy of clinical isolate identification by both the SST method and shortened cultivation was confirmed by MALDI-TOF MS after standard 16–24 h cultivation on solid media. Some isolates could not be reliably identified due to the inability to obtain protein mass spectra.

Following processing of positive blood cultures, preliminary rapid AST was performed using a modified broth microdilution method for both protocols. Although MIC values were determined, our study primarily focused on clinically relevant susceptibility categories to better reflect decision-making in the hospital setting. MICs results were compared with those obtained by standard procedures and interpreted in terms of categorical agreement (CA) and error rates (mE = minor error, ME = major error, VME = very major error).

#### 3.1.1. Direct Identification by SST Method

Among the 135 positive blood cultures processed by the SST method, 63 isolates were Gram-positive. Of these, 28.6% (18/63) were reliably identified at the species level (MALDI score ≥ 2.0), and 53.97% (34/63) were reliably identified at the genus level (MALDI score ≥ 1.7). Using the study-specific cut-off in combination with result consistency, 87.3% (55/63) of Gram-positive isolates were successfully identified. Eight Gram-positive isolates could not be identified, including *C. acnes*, *E. faecium*, *S. aureus*, *S. epidermidis*, *S. haemolyticus*, and *S. pyogenes*.

Among the 135 positive blood cultures, 72 isolates were Gram-negative. Of these, 83.3% (60/72) were reliably identified at the genus and species level (MALDI score ≥ 2.0), and 93.1% (67/72) were reliably identified at the genus level (MALDI score ≥ 1.7). Using the study-specific cut-off in combination with result consistency, a total of 98.6% (71/72) of Gram-negative isolates were successfully identified. One Gram-negative isolate could not be identified, which corresponded to a hypermucoviscous strain *K. pneumoniae*. Table 1 and Table 2 summarize the identification results after SST processing.

#### 3.1.2. Identification After Shortened Cultivation on Solid Media

In this study, bacterial isolates were also identified using MALDI-TOF MS following a shortened incubation on blood agar. Of the total 135 positive blood cultures, 74 isolates were Gram-negative and 61 isolates were Gram-positive. All Gram-negative isolates were reliably identified at both genus and species levels with MALDI scores ≥ 2.0.

Among Gram-positive isolates, 39 (63.93%) were reliably identified at both genus and species levels, and 49 (80.32%) were reliably identified at the genus level with MALDI scores ≥ 1.7. Using our predefined cut-off for identification consistency, a total of 52 Gram-positive isolates (85.24%) were identified. Nine isolates could not be reliably identified following the shortened cultivation, including *E. faecalis*, *M. luteus*, *S. epidermidis*, and *S. hominis*. Table 3 summarizes the identification results after shortened cultivation along with the corresponding MALDI scores.

#### 3.1.3. Determination of Rapid Quantitative Antimicrobial Susceptibility

After identification of bacterial isolate, rapid quantitative AST was performed using a modified broth microdilution method for both experimental protocols. The results obtained were compared with those from the standard workflow. For quality control of AST determination, inoculum density was monitored for both methods in accordance with EUCAST guidelines.

### 3.2. SST Method

Rapid quantitative AST was determined and compared for 108 bacterial isolates, including 64 Gram-negative and 44 Gram-positive isolates. Among the 44 Gram-positive AST results, 37 were for *Staphylococcus* sp., with susceptibility tested against 16 antibiotics. The CA was 99.32%, with mE 0.17% (mE was observed by trimethoprim-sulfamethoxazole) and VME 0.51% (VME were observed by tetracycline and trimethoprim-sulfamethoxazole). *E. faecalis* isolates tested against 10 antibiotics, CA was 98.57% and VME 1.43% (VME was observed by chloramphenicol).

**Figure 2:**
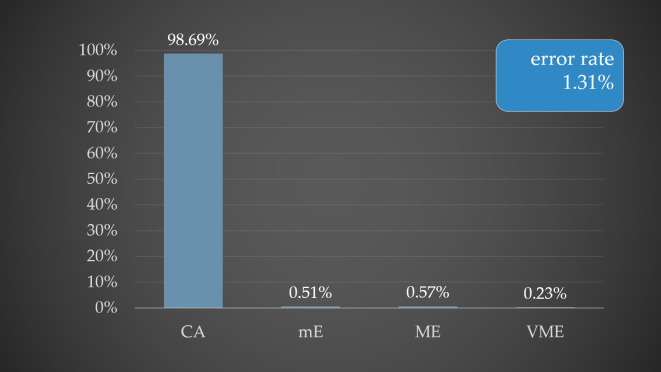
Graphical representation of the percentage distribution of CA and error rates in rapid AST using the SST method.

Among the 64 Gram-negative AST results, 60 were for *Enterobacteriaceae*, tested against 17 antibiotics, showing CA of 98.33%, mE 0.69% (mE were observed by cefepime and ciprofloxacin) and ME 0.98% (ME were observed by ampicillin, ampicillin-sulbactam and tetracycline). For non-fermenting Gram-negative isolates (*P. aeruginosa*), tested against 17 antibiotics, CA was 98.53% and mE 1.47% (mE was observed by imipenem). Overall, this method achieved a CA of 98.69% and an overall error rate of 1.31% (Figure 2).

Figure 2 illustrates the percentages of CA (categorical agreement), minor errors (mE), major errors (ME), and very major errors (VME), which were 98.69%, 0.51%, 0.57%, and 0.23%, respectively, for the SST method. The overall error rate observed for this method was 1.31%.

### 3.3. Shortened Cultivation Method

Rapid quantitative AST was performed and compared for 125 bacterial isolates, including 74 Gram-negative and 51 Gram-positive isolates. Among the 51 Gram-positive AST results, 48 were for *Staphylococcus* sp., tested against 16 antibiotics, with CA 100% and 3 isolates *E. faecalis* tested against 10 antibiotics, CA was 100%.

**Figure 3:**
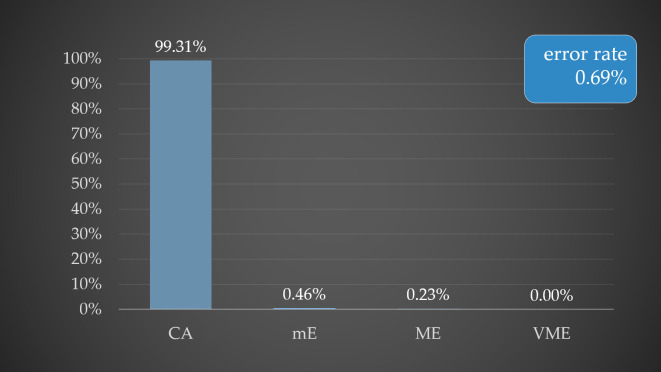
Graphical representation of the percentage distribution of CA and error rates in rapid AST using the shortened cultivation protocol.

Among the 74 Gram-negative AST results, 70 were for *Enterobacteriaceae*, tested against 17 antibiotics, with CA of 98.82%, mE 0.76% (mE were observed by ceftazidime, cefoperazone-sulbactam, cefepime and ciprofloxacin) and ME 0.42% (ME were observed by tobramycin and ciprofloxacin). For non-fermenting Gram-negative isolates (*A. baumannii*), tested against 17 antibiotics, CA showed 100%. Overall, this method achieved a CA of 99.31% and an overall error rate of 0.69% (Figure 3).

Figure 3 shows the percentages of CA, mE, ME, and VME for the shortened cultivation protocol, reaching 99.31%, 0.46%, 0.23%, and 0.00%, respectively. The overall error rate for the shortened cultivation protocol was 0.69%.

## 4. Discussion

BSIs occur when viable microorganisms enter the bloodstream and are not adequately controlled, potentially leading to sepsis and, in severe cases, septic shock with multi-organ failure. BSIs are associated with significant morbidity, mortality, and prolonged hospital stays. Delays in the administration of appropriate antimicrobial therapy can reduce survival in septic patients by approximately 8% per hour. Exactly for this reason, time to positivity (TTP), which influences the course of BSIs, is an important parameter. It is defined as the incubation time from the collection of a blood culture to the reporting of a positive result. This time interval, however, is affected by numerous factors, including patient characteristics, the source of infection, bacterial inoculum size, bacterial species, prior antibiotic therapy, the total volume of blood collected for culture, the type of sample (peripheral blood versus central venous catheter), as well as incubation conditions and transport time to the laboratory [7,8]. Rapid and accurate identification of the causative pathogen and timely initiation of targeted antimicrobial therapy are therefore critical, particularly given the low bacterial load typically present in blood (1–100 CFU/mL). The effectiveness of antimicrobial therapy largely depends on pathogen identification and AST analysis [9]. AST results are essential for guiding therapy, reducing the empirical use of broad-spectrum antibiotics, and monitoring local resistance trends [10]. Studies indicate that indiscriminate use of broad-spectrum antibiotics contributes significantly to increasing antimicrobial resistance, while delayed administration of targeted therapy increases mortality [11].

One of the primary objectives of this study was the direct identification of bacterial isolates from positive blood cultures using MALDI-TOF MS without prior subculture on solid media. First, we used SST method, whereby a total of 135 positive blood culture samples were processed. Using this approach, 57.78% of isolates were identified with a MALDI score ≥ 2.0; and 74.81% with a MALDI score ≥ 1.7. Considering the cut-off established in our study (≥1.5) in conjunction with consistent identification results, 93.33% of isolates were successfully identified. Nine isolates (6.67%) could not be identified. In the study by Azrad et al. (2019) [12], the authors described the use of SST method for rapid identification from positive blood cultures using MALDI-TOF MS. Identification with a MALDI-TOF MS score ≥ 2.0 was achieved for 5.28% of isolates, and 45.5% of isolates were identified with a score ≥ 1.7. Identifications with MALDI-TOF MS scores < 1.7 were not considered in their analysis, and no subsequent ethanol–formic acid extraction was performed prior to MALDI-TOF MS identification. In contrast, in publication [4] authors reported higher identification success rates, with 70.2% of isolates identified at a MALDI-TOF MS score ≥ 2.0 and 92.4% at a score ≥ 1.7. In their study, formic acid was applied to the samples prior to MALDI-TOF MS identification.

Our results demonstrated higher and more reliable identification success for Gram-negative bacteria compared to Gram-positive bacteria. Among the nine unidentified isolates, one was Gram-negative (*K. pneumoniae*) and eight were Gram-positive bacteria. Considering MALDI scores ≥ 1.7; identification success reached 93.05% for Gram-negative isolates and 53.97% for Gram-positive isolates. When applying the cut-off value for identification conjunction with consistent identification result, the identification success rate was 98.61% for Gram-negative bacteria and 87.3% for Gram-positive bacteria. Several studies have reported higher identification success for Gram-negative bacteria using direct MALDI-TOF MS identification [13,14,15,16,17,18]. The lower success rate for Gram-positive bacteria may be attributed to differences in cell wall structure, as thicker cell walls can reduce protein extraction efficiency and, consequently, the quality of mass spectra. Additionally, protein profile similarities among certain Gram-positive species (e.g., streptococci) may impede species-level differentiation [14,15,16,17,18,19].

In some cases, direct MALDI-TOF MS analysis of blood culture samples yielded lower MALDI scores compared to standard subculture-based analysis. Blood components, including serum proteins and cellular debris, can interfere with mass spectrum acquisition, producing background noise and limiting accurate identification [18,20,21,22,23,24]. Sample preparation protocols, including erythrocyte lysis, serum protein removal, and purification from culture medium components, are essential for reliable results [18,25,26,27].

We also evaluated bacterial identification after shortened cultivation (4 h) on solid media. Among 135 positive blood cultures, 74 Gram-negative isolates were identified with 100% success at genus and species level. For Gram-positive isolates, identification success was lower: 63.93% at species level, 80.33% at genus level, and 85.25% considering to our cut-off in conjunction with consistency. Nine Gram-positive isolates could not be identified. Previous studies have reported variable identification success following shortened cultivation, depending on species, growth rate, culture media, and incubation time [28,29]. Proper application of immature bacterial biomass to the MALDI target plate is also a critical factor.

When the identification cut-off established in this study was applied in combination with result consistency, the identification success rate using the SST method increased by 38.89% (5.56% for Gram-negative bacteria and 33.33% for Gram-positive bacteria). For shortened cultivation, the increase was 4.92% and was observed only for Gram-positive bacteria. All rapid identification results were confirmed by the standard identification procedure and showed complete concordance. Several studies have reported the use of lower MALDI-TOF MS score thresholds to improve identification success. Moussaoui et al. (2010) [30] applied an identification cut-off of >1.4 for bacterial isolates when result consistency was observed in at least four consecutive identification proposals and the highest MALDI score exceeded 1.4. In that study, the use of a lower score threshold than recommended by the manufacturer increased the success rate of direct identification by 5.2%. Zengin Canalp and Bayraktar [4] also reported higher identification success when a lower MALDI-TOF MS score cut-off was applied (MALDI score > 1.5).

Following direct identification, we performed rapid quantitative AST using a modified broth microdilution method, comparing two protocols: SST-derived bacterial pellets and short-term cultivation. Method with bacterial pellet (SST method) achieved CA 98.69% in comparison with standard AST results, mE was 0.51%, ME 0.57% and VME 0.23%. Several studies have published results of direct AST determination from positive blood cultures using various automated AST systems [31,32,33,34]. For example, Beuving et al. (2011) [31] also processed positive blood culture samples using the SST method for subsequent AST determination. In their study, AST was performed using the BD Phoenix system, achieving a CA of 99% for Gram-negative bacteria, with mE 0.7% and VME 0.3%. In contrast, for Gram-positive bacteria, CA was 95.4%, with mE, ME and VME rates of 1.1%, 3.1%, and 0.4%, respectively. On the other side in our study, the CA for the SST method for Gram-negative bacteria was 98.3%, with mE 0.7% and ME 0.9%. For Gram-positive bacteria, the CA was 99.2%, with mE rate of 0.2% and a VME rate of 0.6%. The CA for Gram-positive bacteria was higher compared with that for Gram-negative bacteria; however, conversely, a VME rate of 0.6% was observed in Gram-positive isolates. In the present study, VME were observed exclusively with the protocol employing the SST method. In staphylococci, these discrepancies were associated with AST results for tetracycline and trimethoprim–sulfamethoxazole. Additionally, a VME was identified for chloramphenicol in *E. faecalis*.

In the case of the second method, shortened cultivation, we performed rapid quantitative AST from immature bacterial biomass after shortened cultivation. This method achieved CA 99.31% in comparison with standard AST results, mE was 0.46% and ME 0.23%. Using this method, the CA for Gram-positive bacteria was 100%, and the CA for Gram-negative bacteria was 98.9%; the mE was 0.7% and the ME was 0.4%.

Based on the obtained results, the overall error rate was lower for AST performed after shortened incubation (error rate: 0.69%) compared with AST performed from bacterial cell pellets obtained using the SST method (error rate: 1.31%). The bacterial inoculum obtained during processing of bacterial cell pellets using the SST method did not, in some cases, meet the required inoculum density for AST determination. This may happen due to the presence of non-bacterial blood components in samples from positive blood cultures. Residual blood components may subsequently contribute to increased turbidity during AST processing, potentially affecting overall rapid AST determination and the observed error rate.

The rapid identification and rapid AST methods have inherent limitations. One major limitation is the presence of multiple pathogens in a single positive blood culture bottle [35]. For the SST method, identification depends on direct analysis of blood samples and the acquisition of adequate protein mass spectra, whereas for the shortened culture protocol, it relies on the growth rate of individual bacterial isolates on solid media. Similarly, rapid AST requires a sufficient bacterial pellet in the SST method or adequate growth in the shortened culture protocol to achieve the appropriate bacterial turbidity (0.5 McFarland). Additionally, the method is dependent on the bacterial species, as certain pathogens—such as *Enterococcus faecium*, anaerobic bacteria, and some streptococci—are not included in the Bel-MIDITECH (Slovakia) diagnostic AST panel and therefore cannot be tested using the rapid AST approach.

The methods employed in this study (rapid identification and AST) are not financially burdensome for routine laboratory practice. Identification using MALDI-TOF MS, which is already widely available in most clinical microbiology laboratories, involves in our setting only the cost of the SST tube. The shortened cultivation protocol does not require any additional consumables. The costs associated with AST are limited to the Bel-MIDITECH diagnostic AST panel and reagents, which is routinely used in our laboratory for routine AST.

Overall, the high rate of successful bacterial isolate identification and the high success rate of rapid AST suggest that the obtained results could be beneficial for the timely initiation of appropriate antibiotic therapy or for early modification of treatment, including treatment de-escalation, which ultimately contributes to improved patient prognosis.

## 5. Conclusions

BSIs are associated with high morbidity and mortality, making rapid pathogen identification and timely targeted antimicrobial therapy essential. In this study, MALDI-TOF MS enabled rapid identification of bacterial pathogens directly from positive blood cultures using the SST method, as well as after shortened incubation on solid media. Both approaches proved suitable for accelerating pathogen identification, although limitations were observed in cases where adequate protein spectra could not be obtained. The main advantage of these rapid protocols is the ability to report pathogen identification on the same day as blood culture positivity—within approximately 1 h using the SST method and after at least 4 h of shortened cultivation, depending on the species. In addition, implementation of rapid AST reduced the time to susceptibility results by approximately 24 h, facilitating earlier targeted therapy and supporting antimicrobial stewardship. Nevertheless, conventional culture methods and quantitative AST remain essential for definitive microbiological diagnosis and therapeutic decision-making.

## Figures and Tables

**Table 1:** Summary identification table of Gram-positive bacterial isolates using the SST method. The table presents the identification results of Gram-positive isolates obtained by the SST method (*n* = 63), including the achieved MALDI-TOF MS score values ≥2.0 (*n* = 18), 1.7–1.99 (*n* = 16) and ≥1.5–1.699 (*n* = 21) or cases classified as NOIP (*n* = 8). Furthermore, the table shows the percentage distribution of isolates within the respective MALDI-TOF MS identification score ranges.

Identification of Gram-Positive Bacteria	≥2.0	1.7–1.99	≥1.5–1.699	NOIP
*Actinomyces oris*			1	
*Bacillus species*	1		1	
*Cutibacterium acnes*				2
*Granulicatella adiacens*	1			
*Enterococcus faecalis*	1	3	3	
*Enterococcus faecium*		1	2	1
*Parvimonas micra*			1	
*Staphylococcus aureus*	7	6	5	2
*Staphylococcus capitis*	1			
*Staphylococcus epidermidis*	2	1	2	1
*Staphylococcus haemolyticus*		1	1	1
*Staphylococcus hominis*	3	1	3	
*Streptococcus agalactiae*	1	2		
*Streptococcus anginosus*			1	
*Streptococcus pyogenes*	1	1	1	1
	18/63 **28.57%**	16/63 **25.40%**	21/63 **33.33%**	8/63 **12.70%**

**Table 2:** Summary identification table of Gram-negative bacterial isolates using the SST method. The table presents the identification results of Gram-negative isolates obtained by the SST method (*n* = 72), including the achieved MALDI-TOF MS score values ≥2.0 (*n* = 60), 1.7–1.99 (*n* = 7) and ≥1.5–1.699 (*n* = 4) or cases classified as NOIP (*n* = 1). Furthermore, the table shows the percentage distribution of isolates within the respective MALDI-TOF MS identification score ranges.

Identification of Gram-Negative Bacteria	≥2.0	1.7–1.99	≥1.5–1.699	NOIP
*Bacteroides fragilis*	3			
*Citrobacter koseri*	2			
*Fusobacterium* *necrophorum*	2			
*Escherichia coli*	28	1	2	
*Haemophillus influenzae*		1		
*Klebsiella oxytoca*	1			
*Klebsiella pneumoniae*	14	4	1	1
*Morganella morganii*	1			
*Pasteurella multocida*	2			
*Proteus mirabilis*	2		1	
*Pseudomonas aeruginosa*	3	1		
*Salmonella species*	2			
	60/72	7/72	4/72	1/72
	**83.33%**	**9.72%**	**5.56%**	**1.39%**

**Table 3:** Summary identification table of Gram-positive and Gram-negative bacterial isolates using shortened cultivation. The table presents in the first part the identification results of Gram-positive isolates obtained by method of shortened cultivation (*n* = 61), including the achieved MALDI-TOF MS score values ≥2.0 (*n* = 39), 1.7–1.99 (n = 10) and ≥1.5–1.699 (*n* = 3) or cases classified as NOIP (*n* = 9). In the second part, the table presents the identification results of Gram-negative isolates (*n* = 74), including the achieved MALDI-TOF MS score values ≥2.0 (*n* = 74). Furthermore, the table shows the percentage distribution of isolates within the respective MALDI-TOF MS identification score ranges.

**Identification of Gram-Positive Bacteria**	**≥2.0**	**1.7–1.99**	**≥1.5–1.699**	**NOIP**
*Enterococcus faecalis*	3			1
*Enterococcus faecium*	1			
*Micrococcus luteus*				2
*Staphylococcus aureus*	25	1		
*Staphylococcus capitis*		1		
*Staphylococcus haemolyticus*		3	2	
*Staphylococcus hominis*	7	3		3
*Staphylococcus epidermidis*	3	2	1	3
	39/61 **63.93%**	10/61 **16.39%**	3/61 **4.92%**	9/61 **14.75%**
**Identification of Gram-Negative Bacteria**	**≥2.0**	**1.7–1.99**	**≥1.5–1.699**	**NOIP**
*Acinetobacter baumanii*	2			
*Citrobacter freundii*	2			
*Escherichia coli*	34			
*Klebsiella pneumoniae*	19			
*Klebsiella aerogenes*	2			
*Morganella morganii*	1			
*Pasteurella multocida*	2			
*Proteus mirabilis*	7			
*Serratia marecescens*	5			
	74/74 **100.00%**			

## Data Availability

The original contributions presented in the study are included in the article; further inquiries can be directed to the corresponding authors.

## Appendix A

Overview of the tested antibiotics and the range of concentrations evaluated [mg/L] using the diagnostic AST kit from Bel-MIDITECH s.r.o. (Slovakia).

**Table A1:** The table includes a panel of antibiotics with the range of concentrations [mg/L] tested against *E. faecalis*.

List of Antibiotics for *E. faecalis*	Range of Concentrations [mg/L]
Ampicillin	0.25–32
Piperacillin-tazobactam	0.5 + 4–64 + 4
Teicoplanin	0.125–16
Vancomycin	0.125–16
Gentamicin	0.25–128
Chloramphenicol	0.25–32
Ciprofloxacin	0.03–4
Tetracycline	0.125–16
Trimethoprim-sulfamethoxazole	0.1 + 2.5–4 + 76
Nitrofurantoin	2–64

**Table A2:** The table includes a panel of antibiotics with the range of concentrations [mg/L] tested against *Enterobacteriaceae*.

List of Antibiotics for *Enterobacteriaceae*	Range of Concentrations [mg/L]
Ampicillin	0.25–32
Ampicillin-sulbactam	0.2 + 0.1–32 + 4
Piperacillin-tazobactam	0.5 + 4–64 + 4
Cefuroxime	0.25–32
Cefotaxime	0.25–32
Cefotaxime + clavulanic acid	0.25 + 4–32 + 4
Ceftazidime	0.25–32
Ceftazidime + clavulanic acid	0.25 + 4–32 + 4
Cefoperazone + sulbactam	0.5 + 0.2–64 + 26
Cefepime	0.25–32
Ertapenem	0.03–4
Meropenem	0.125–16
Gentamicin	0.125–16
Tobramycin	0.125–16
Amikacin	0.5–64
Tigecycline	0.03–4
Ciprofloxacin	0.03–4
Tetracycline	0.125–16
Colistin	0.25–8
Tripmethoprim + sulfamethoxazole	0.125 + 2–4 + 76

**Table A3:** The table includes a panel of antibiotics with the range of concentrations [mg/L] tested against Gram-negative non-fermenting bacteria.

List of Antibiotics for Gram-Negative Non-Fermenting Bacteria	Range of Concentrations [mg/L]
Ampicillin-sulbactam	0.2 + 0.1–32 + 16
Piperacillin	0.5–64
Piperacillin-tazobactam	0.5 + 4–64 + 4
Cefotaxime	0.25–32
Cefotaxime + clavulanic acid	0.25 + 4–32 + 4
Ceftazidime	0.25–32
Ceftazidime + clavulanic acid	0.25 + 4–32 + 4
Cefoperazone + sulbactam	0.5 + 0.2–64 + 26
Cefepime	0.25–32
Aztreonam	0.25–32
Imipenem	0.25–32
Meropenem	0.125–16
Gentamicin	0.125–16
Tobramycin	0.125–16
Amikacin	0.5–64
Chloramphenicol	0.25–32
Ciprofloxacin	0.03–4
Tetracycline	0.125–16
Colistin	0.25–8
Tripmethoprim + sulfoamethoxazole	0.125 + 2–4 + 76

**Table A4:** The table includes a panel of antibiotics with the range of concentrations [mg/L] tested against Gram-positive bacteria (staphylococci).

List of Antibiotics for Gram-Positive Bacteria (Staphylococci)	Range of Concetrations [mg/L]
Ampicillin	0.25–32
Ampicillin-sulbactam	0.2 + 4–32 + 4
Oxacillin	0.03–4
Cefoxitin	0.125–16
Piperacillin-tazobactam	0.5 + 4–64 + 4
Erythromycin	0.06–8
Clindamycin	0.03–4
Linezolid	0.06–8
Rifampicin	0.03–4
Gentamicin	0.25–128
Teicoplanin	0.125–16
Vancomycin	0.125–16
Trimethoprim	0.125–16
Chloramphenicol	0.25–32
Tigecycline	0.03–4
Moxifloxacin	0.03–4
Ciprofloxacin	0.03–4
Tetracycline	0.125–16
Tripmethoprim + sulfamethoxazole	0.125 + 2–4 + 76
Nitrofurantoin	2–64

## References

[1] Kumar A., Roberts D., Wood K.E., Light B., Parrillo J.E., Sharma S., Suppes R., Feinstein D., Zanotti S., Taiberg L. (2006). Duration of hypotension before initiation of effective antimicrobial therapy is the critical determinant of survival in human septic shock. Crit. Care Med..

[2] Rhee C., Dantes R., Epstein L., Murphy D.J., Seymour C.W., Iwashyna T.J., Kadri S.S., Angus D.C., Danner R.L., Fiore A.E. (2017). Incidence and trends of sepsis in US hospitals using clinical vs claims data, 2009–2014. JAMA.

[3] Khan S., Das A., Mishra A., Vidyarthi A.J., Nandal M., Yadav H., Roy S., Singh M. (2024). Evaluation of three protocols for direct susceptibility testing for gram-negative rods from flagged positive blood culture bottles. Microbiol. Spectr..

[4] Zengin Canalp H., Bayraktar B. (2021). Direct rapid identification from positive blood cultures by MALDI-TOF MS: Specific focus on turnaround times. Microbiol. Spectr..

[5] Singhal N., Kumar M., Kanaujia P.K., Virdi J.S. (2015). MALDI-TOF mass spectrometry: An emerging technology for microbial identification and diagnosis. Front. Microbiol..

[6] Idelevich E.A., Becker K. (2019). How to accelerate antimicrobial susceptibility testing. Clin. Microbiol. Infect..

[7] Lamy B. (2019). Blood culture time-to-positivity: Making use of the hidden information. Clin. Microbiol. Infect..

[8] Blot F., Schmidt E., Nitenberg G., Tancrède C., Leclercq B., Laplanche A., Andremont A. (1998). Earlier positivity of central-venous- versus peripheral-blood cultures is highly predictive of catheter-related sepsis. J. Clin. Microbiol..

[9] Tjandra K.C., Ram-Mohan N., Abe R., Hashemi M.M., Lee J.H., Chin S.M., Roshardt M.A., Liao J.C., Wong P.K., Yang S. (2022). Diagnosis of bloodstream infections: An evolution of technologies towards accurate and rapid identification and antibiotic susceptibility testing. Antibiotics.

[10] Quirino A., Marascio N., Peronace C., Gallo L., Barreca G.S., Giancotti A., Lamberti A.G., Colosimo M., Minchella P., Trecarichi E.M. (2021). Direct antimicrobial susceptibility testing (AST) from positive blood cultures using Microscan system for early detection of bacterial resistance phenotypes. Diagn. Microbiol. Infect. Dis..

[11] Shi X., Sharma S., Chmielewski R.A., Markovic M.J., VanEpps J.S., Yau S.T. (2024). Rapid diagnosis of bloodstream infections using a culture-free phenotypic platform. Commun. Med..

[12] Azrad M., Keness Y., Nitzan O., Pastukh N., Tkhawkho L., Freidus V., Peretz A. (2019). Cheap and rapid in-house method for direct identification of positive blood cultures by MALDI-TOF MS technology. BMC Infect. Dis..

[13] Barberino M.G., Silva M.O., Arraes A.C.P., Correia L.C., Mendes A.V. (2017). Direct identification from positive blood broth culture by matrix-assisted laser desorption-ionization time-of-flight mass spectrometry (MALDI-TOF MS). Braz. J. Infect. Dis..

[14] Zhou M., Yang Q., Kudinha T., Sun L., Zhang R., Liu C., Yu S., Xiao M., Kong F., Zhao Y. (2017). An improved in-house MALDI-TOF MS protocol for direct cost-effective identification of pathogens from blood cultures. Front. Microbiol..

[15] Homolová R., Bogdanová K., Bardoň J., Kolář M. (2020). Direct identification of bacteria in blood cultures by MALDI-TOF MS. Klin. Mikrobiol. Infekc. Lek..

[16] Huang Y.L., Sun Q.L., Li J.P., Hu Y.Y., Zhou H.W., Zhang R. (2019). Evaluation of an in-house MALDI-TOF MS rapid diagnostic method for direct identification of micro-organisms from blood cultures. J. Med. Microbiol..

[17] Chien J.Y., Lee T.F., Du S.H., Teng S.H., Liao C.H., Sheng W.H., Teng L.J., Hsueh P.R. (2016). Applicability of an in-house saponin-based extraction method in Bruker Biotyper matrix-assisted laser desorption/ionization time-of-flight mass spectrometry system for identification of bacterial and fungal species in positively flagged blood cultures. Front. Microbiol..

[18] La Scola B., Raoult D. (2009). Direct identification of bacteria in positive blood culture bottles by matrix-assisted laser desorption ionisation time-of-flight mass spectrometry. PLoS ONE.

[19] Wang J., Wang H., Cai K., Yu P., Liu Y., Zhao G., Chen R., Xu R., Yu M. (2021). Evaluation of three sample preparation methods for the identification of clinical strains by using two MALDI-TOF MS systems. J. Mass Spectrom..

[20] Simon L., Ughetto E., Gaudart A., Degand N., Lotte R., Ruimy R. (2019). Direct identification of 80 percent of bacteria from blood culture bottles by matrix-assisted laser desorption ionization-time of flight mass spectrometry using a 10-minute extraction protocol. J. Clin. Microbiol..

[21] Ferroni A., Suarez S., Beretti J.L., Dauphin B., Bille E., Meyer J., Bougnoux M.E., Alanio A., Berche P., Nassif X. (2010). Real-time identification of bacteria and *Candida* species in positive blood culture broths by matrix-assisted laser desorption ionization-time of flight mass spectrometry. J. Clin. Microbiol..

[22] Opota O., Croxatto A., Prod’hom G., Greub G. (2015). Blood culture-based diagnosis of bacteraemia: State of the art. Clin. Microbiol. Infect..

[23] Nomura F., Tsuchida S., Murata S., Satoh M., Matsushita K. (2020). Mass spectrometry-based microbiological testing for blood stream infection. Clin. Proteom..

[24] Murray P.R., Masur H. (2012). Current approaches to the diagnosis of bacterial and fungal bloodstream infections in the intensive care unit. Crit. Care Med..

[25] Tanner H., Evans J.T., Gossain S., Hussain A. (2017). Evaluation of three sample preparation methods for the direct identification of bacteria in positive blood cultures by MALDI-TOF. BMC Res. Notes.

[26] Loonen A.J., Jansz A.R., Stalpers J., Wolffs P.F., van den Brule A.J. (2012). An evaluation of three processing methods and the effect of reduced culture times for faster direct identification of pathogens from BacT/ALERT blood cultures by MALDI-TOF MS. Eur. J. Clin. Microbiol. Infect. Dis..

[27] Schubert S., Weinert K., Wagner C., Gunzl B., Wieser A., Maier T., Kostrzewa M. (2011). Novel, improved sample preparation for rapid, direct identification from positive blood cultures using matrix-assisted laser desorption/ionization time-of-flight (MALDI-TOF) mass spectrometry. J. Mol. Diagn..

[28] Froböse N.J., Idelevich E.A., Schaumburg F. (2021). Short incubation of positive blood cultures on solid media for species identification by MALDI-TOF MS: Which agar is the fastest?. Microbiol. Spectr..

[29] Demir M., Hazırolan G. (2024). Rapid bacterial identification from positive blood cultures by MALDI-TOF MS following short-term incubation on solid media. Infect. Dis. Clin. Microbiol..

[30] Moussaoui W., Jaulhac B., Hoffmann A.M., Ludes B., Kostrzewa M., Riegel P., Prévost G. (2010). Matrix-assisted laser desorption ionization time-of-flight mass spectrometry identifies 90% of bacteria directly from blood culture vials. Clin. Microbiol. Infect..

[31] Beuving J., van der Donk C.F., Linssen C.F., Wolffs P.F., Verbon A. (2011). Evaluation of direct inoculation of the BD PHOENIX system from positive BACTEC blood cultures for both Gram-positive cocci and Gram-negative rods. BMC Microbiol..

[32] Kumar M., Shergill S.P.S., Tandel K., Sahai K., Gupta R.M. (2019). Direct antimicrobial susceptibility testing from positive blood culture bottles in laboratories lacking automated antimicrobial susceptibility testing systems. Med. J. Armed Forces India.

[33] Khanal P.C., Richardson J.C., Richardson K.G., Damhorst G.L., Oertell O.J., Filbrun A., Burd E.M., Dickson R.M. (2025). Rapid colorimetric antimicrobial susceptibilities direct from positive blood culture for Gram-negative bacteria. Microbiol. Spectr..

[34] Morales P., Tang P., Mariano E., Gopalan A., Aji N., Pérez-López A., Suleiman M. (2024). Evaluation of Direct Antimicrobial Susceptibility Testing of Gram-Negative Bacilli and *Staphylococcus aureus* from Positive Pediatric Blood Culture Bottles Using BD Phoenix M50. Microorganisms.

[35] Samuel L. (2023). Direct-from-Blood Detection of Pathogens: A Review of Technology and Challenges. J. Clin. Microbiol..

